# The effect of phenobarbital treatment on behavioral comorbidities and on the composition and function of the fecal microbiome in dogs with idiopathic epilepsy

**DOI:** 10.3389/fvets.2022.933905

**Published:** 2022-08-04

**Authors:** Antja Watanangura, Sebastian Meller, Jan S. Suchodolski, Rachel Pilla, Mohammad R. Khattab, Shenja Loderstedt, Lisa F. Becker, Andrea Bathen-Nöthen, Gemma Mazzuoli-Weber, Holger A. Volk

**Affiliations:** ^1^Department of Small Animal Medicine and Surgery, University of Veterinary Medicine Hannover, Hannover, Germany; ^2^Center for Systems Neuroscience (ZSN), Hannover, Germany; ^3^Veterinary Research and Academic Service, Faculty of Veterinary Medicine, Kasetsart University, Kamphaeng Saen, Nakhon Pathom, Thailand; ^4^Gastrointestinal Laboratory, Department of Small Animal Clinical Sciences, Texas A&M University, College Station, TX, United States; ^5^Department for Small Animal, Faculty of Veterinary Medicine, Leipzig University, Leipzig, Germany; ^6^Tierarztpraxis, Dr A. Bathen-Nöthen, Cologne, Germany; ^7^Institute for Physiology and Cell Biology, University of Veterinary Medicine Hannover, Hannover, Germany

**Keywords:** canine idiopathic epilepsy, phenobarbital, gastrointestinal microbiota, short-chain fatty acids, butyrate, behavioral comorbidities

## Abstract

Phenobarbital (PB) is one of the most important antiseizure drugs (ASDs) to treat canine idiopathic epilepsy (IE). The effect of PB on the taxonomic changes in gastrointestinal microbiota (GIM) and their functions is less known, which may explain parts of its pharmacokinetic and pharmacodynamic properties, especially its antiseizure effect and drug responsiveness or drug resistance as well as its effect on behavioral comorbidities. Fecal samples of 12 dogs with IE were collected prior to the initiation of PB treatment and 90 days after oral PB treatment. The fecal samples were analyzed using shallow DNA shotgun sequencing, real-time polymerase chain reaction (qPCR)-based dysbiosis index (DI), and quantification of short-chain fatty acids (SCFAs). Behavioral comorbidities were evaluated using standardized online questionnaires, namely, a canine behavioral assessment and research questionnaire (cBARQ), canine cognitive dysfunction rating scale (CCDR), and an attention deficit hyperactivity disorder (ADHD) questionnaire. The results revealed no significant changes in alpha and beta diversity or in the DI, whereas only the abundance of Clostridiales was significantly decreased after PB treatment. Fecal SCFA measurement showed a significant increase in total fecal SCFA concentration and the concentrations of propionate and butyrate, while acetate concentrations revealed an upward trend after 90 days of treatment. In addition, the PB-Responder (PB-R) group had significantly higher butyrate levels compared to the PB-Non-Responder (PB-NR) group. Metagenomics of functional pathway genes demonstrated a significant increase in genes in trehalose biosynthesis, ribosomal synthesis, and gluconeogenesis, but a decrease in V-ATPase-related oxidative phosphorylation. For behavioral assessment, cBARQ analysis showed improvement in stranger-directed fear, non-social fear, and trainability, while there were no differences in ADHD-like behavior and canine cognitive dysfunction (CCD) scores after 90 days of PB treatment. While only very minor shifts in bacterial taxonomy were detected, the higher SCFA concentrations after PB treatment could be one of the key differences between PB-R and PB-NR. These results suggest functional changes in GIM in canine IE treatment.

## Introduction

Idiopathic epilepsy (IE) is a common neurological disease in dogs, with an unknown underlying cause and no identifiable structural brain abnormalities (1). IE is typically treated lifelong with antiseizure drugs (ASDs) (2). Phenobarbital (PB) is an ASD that is used as the primary treatment of choice for canine IE due to its widespread availability, tolerance, and affordability (2–4). It mainly acts on the allosteric site of gamma-aminobutyric acid subtype A (GABA_A_) receptors, causing the prolongation of its receptor channel opening, which enhances response to inhibitory neurotransmitter gamma-aminobutyric acid (GABA) or in higher concentrations, open the GABA channel alone, causing neuronal hyperpolarization (5). However, some dogs with IE continue to have a seizure despite PB treatment, which is referred to as “PB resistance” (6). The role of the microbiota–gut–brain axis (MGBA) in PB or drug-resistant epilepsy has not yet been fully explored.

MGBA is a bidirectional communication system between the enteric microbiota and the brain, which mainly consists of the central nervous system (CNS), enteric nervous system (ENS), and gastrointestinal microbiota (GIM) (7, 8). Communication is exerted through many pathways, namely, neural tracts, immunological, inflammatory, and neuroendocrine pathways (9). In the last decade, scientific evidence has increased substantially, highlighting the relation between GIM and neuropsychiatric and neurological disorders, namely, epilepsy (10–12). The first milestone in epilepsy was made by a preclinical study in mice by Olson and colleagues, which showed specific GIM to be responsible for increasing seizure threshold (13). This study led to an increased interest in MGBA relating to epilepsy and its role in treatment response.

In human medicine, patients with epilepsy differ in their GIM from healthy controls (14). Moreover, a difference in the GIM between human patients with drug-sensitive and drug-resistant epilepsy was also noted. A study found that the GIM composition of drug-sensitive epilepsy was similar to that of healthy people, while the GIM of patients with drug-resistant epilepsy showed an increased abundance of rare bacteria (11). In veterinary medicine, a study showed a significant reduction in GABA and short-chain fatty acids (SCFAs) producing bacteria in dogs with epilepsy compared to healthy controls (15). Another study in canine IE showed a change in microbiota composition when treating dogs with a medium-chain triglyceride-enriched diet, a diet used to improve seizure control (16).

Normally, GIM diversity and their functions can be affected to varying degrees by several factors, such as diet, environment, disease, and medication (17, 18). One of the main metabolites of GIM is SCFAs. SCFAs are fatty acids with fewer than six carbons produced by bacteria, such as Bifidobacterium, Clostridium, and Lactobacillus during polysaccharide fermentation in the colon (19, 20). The main SCFAs produced by GIM are acetate, propionate, and butyrate, which are absorbed through the intestine before being circulated to the liver and other organs, namely, the brain (9, 20). SCFAs play a major role in maintaining a healthy intestine as they are the main energy source of enterocytes and promote intestinal barrier integrity (21, 22). Furthermore, SCFAs work *via* MGBA, providing their anti-inflammatory, immunomodulatory, and neuroprotective abilities (23–25).

Like in people, fear- and anxiety-related behaviors, as well as cognitive dysfunctions, have been reported in treated and untreated dogs with IE (26–32). To date, it has not yet been completely elucidated whether these epilepsy comorbidities are a risk factor for the development of epilepsy, part of the epilepsy phenotype, a side effect of some ASDs, or a combination of all three factors. Therefore, the aim of the current study was to further investigate the effect of PB on behavior and on the composition and function of GIM as well as their association to PB response in dogs with IE. The results of this study could lead to a better understanding of the association of GIM and their function in canine IE and epilepsy comorbidities.

## Materials and methods

### Dogs

Owners of drug-naïve dogs with IE [Tier II confidence level (33)] were recruited and first informed about the current standard of care for IE management (2). Then, only dogs were included in the study for which the owners had given formal consent to initiate PB treatment. Only one dog per household was allowed to be enrolled. Diets were recorded and not modified, and the owners were told not to change the diet throughout the study period.

### Phenobarbital treatment

The initial PB dose given was based on the International Veterinary Epilepsy Task Force (IVETF) consensus statement: 2.5 mg/kg orally every 12 h (34). The dose of PB was not changed during the 90-day study period. On days 30 and 90, the PB serum concentrations were measured.

### Fecal samples

The fecal samples were collected prior to PB treatment (D0) and 90 days after continuous PB treatment (D90). The samples were stored in a plastic tube (5 ml, 57 × 15.3 mm, polypropylene; Sarstedt AG & Co. KG, Nümbrecht, Germany) at −80°C before being shipped with dried ice to the Gastrointestinal Laboratory of Texas A&M University, College Station, Texas, USA, for qPCR-based dysbiosis index (DI) and SCFA analysis, and further delivered to Diversigen, Inc., Houston, TX, USA for metagenomics by shallow DNA shotgun sequencing.

### Shallow DNA shotgun sequencing (metagenomics)

The microbial DNA from fecal samples was extracted and quantified using the MoBio PowerSoil® DNA isolation kit (MoBio Laboratories, Carlsbad, CA, USA) and the Quant-iT PicoGreen dsDNA assay kit (Thermo Fisher Scientific Inc., Waltham, MA, USA), respectively. For sequencing libraries preparation, the Nextera XT DNA Library Preparation Kit (Illumina Inc., San Diego, CA, USA) was used before the libraries were pooled. After this step, SPRI bead purification and concentration were processed using SpeedBeads Magnetic Carboxylate Modified Particles (Cytiva Life Sciences, Marlborough, MA, USA). The resulting pooled libraries were denatured by NaOH before being diluted and spiked by 2% PhiX. The metagenomic sequencing was performed on an Illumina NextSeq 500 System using NextSeq 500/550 High Output 150 cycle kit (1 x 145 bp reads), followed by being multiplexed on the sequencer before converting to FASTQ files and filtering for low quality (Q-score <30) and length (<50). Adapter sequences were trimmed, and all sequences were trimmed to a maximum length of 100 bp prior to alignment. The raw sequences were made using NCBI Sequence Read Archive before analysis with established pipelines. In terms of taxonomic classification, FASTA sequences were aligned making a curated database, which contained all representative genomes in the NCBI RefSeq representative genome collection for prokaryotes (release 86) with additional manually curated strains for bacteria (35). Alignments were made at 97% identity and compared to reference genomes. The input sequences were listed for taxonomy assignment as the lowest common ancestor, which was compatible with not <80% of the reference sequences. OTUs accounting for < one million of all species-level markers and OTUs with <0.01% of their unique genome regions matching as well as <0.1% of the whole genome were discarded. For downstream analysis, normalized and filtered tables were used in QIIME2.

Alpha diversity was evaluated by the number of species, Shannon–Wiener index, Pielou's index, and observed operational taxonomic units (OTUs) using a rarefied OTU table. Beta diversity was evaluated by weighted and unweighted UniFrac distance measures, and principal coordinate analysis (PCoA) plots using the Bray–Curtis dissimilarity.

The differences between each GIM community were investigated by Bray–Curtis distance metric analysis and beta diversity with QIIME2. Kyoto Encyclopedia of Genes and Genomes (KEGG) orthology (KO) groups were observed with alignment at 97% identity against a gene database derived from the NCBI RefSeq representative genome collection for prokaryotes with additional manually curated strains for bacteria mentioned above for functionally annotated genes (36, 37). The directly observed KO counts reported as relative abundance within each sample were expressed in a KO table and downstream tables. KOs were then collapsed to level-2 and level-3 KEGG pathways and KEGG modules.

### Fecal dysbiosis index and quantitative real-time PCR

All fecal samples were analyzed, and DNA was extracted from 100 mg of each fecal sample using a MoBio PowerSoil® DNA isolation kit (MoBio Laboratories, Carlsbad, CA, USA). The total bacteria and specific bacterial taxa (i.e., *Faecalibacterium, Turicibacter, Streptococcus, Escherichia coli, Blautia, Fusobacterium, Clostridium hiranonis*, and *Bifidobacterium*) were analyzed using Quantitative PCR assays (qPCR). The PCR conditions were performed in the following order: at 95°C maintained for 2 min, 40 cycles at 95°C for 5 s, and then annealing for 10 s at the optimized temperature using 10 μL of SYBR-based reaction mixtures (5 μL of SsoFast™ EvaGreen® supermix [Bio-Rad Laboratories GmbH, Düsseldorf, Germany]), 1.6 μL of high-quality PCR water, 0.4 μL of each primer (final concentration: 400 nM), 1 μL of 1% BSA (final concentration: 0.1%), and 2 μL of DNA (1:10 or 1:100 dilution). The qPCR results were reported in log amount of microbial DNA (fg) for each taxon per 10 ng of isolated total DNA. The DI was calculated combined with qPCR assay results. The DI at 0 was used as a cut-off for normobiosis (DI <0) (38).

### SCFAs analysis

The concentration of SCFAs, namely, acetate, propionate, butyrate, isobutyrate, valerate, and isovalerate was analyzed by stable isotope dilution gas chromatography-mass spectrometry (GC-MS) assay [modified from (39)]. The fecal samples from each time point were thawed at room temperature and then vortexed for 30 min before being centrifuged for 20 min at 2,100 g at 4°C. The fecal supernatants from each sample were separated using serum filters (Fisherbrand serum filter system, Fisher Scientific Inc., Pittsburgh, PA, USA). The amount of 500 μL of each supernatant sample was mixed with 10 μL of internal standard (200 mM heptadeuterated butyric acid). A C18 solid-phase extraction column (Sep-Pak C18 1 cc Vac Cartridge, Waters Corporation, Milford, MA, USA) was then used to extract the mixtures. The samples were derivatized by N-tert-butyldimethylsilyl-N-methyltrifluoroacetamide (MTBSTFA) for 60 min at room temperature. The chromatographic separation and quantification of the processed samples were performed using a GC (Agilent 6890N, Agilent Technologies Inc., Santa Clara, CA, USA) connected to an electron ionizing MS (Agilent 5975C, Agilent Technologies Inc.). A DB-1 ms capillary column (Agilent Technologies Inc.) was used for separation. The separation was performed under the following temperature program: 40°C maintained for 0.1 min, increased by 5°C/min to 70°C, then maintained for 3.5 min, increased by 20°C/min to 160°C, and lastly increased by 35°C/min to 280°C and maintained for 3 min. This process lasted for 20.53 min. The MS in electron impact positive-ion mode was used with selective ion monitoring at mass-to-charge ratios (M/Z) of 117 (acetate), 131 (propionate), 145 (butyrate), and 152 (heptadeuterated butyrate; internal standard). In terms of quantification, the ratio was calculated using the area under curve of the internal standard and each SCFA. The minimum detection limits of acetate, propionate, and butyrate concentrations were 1.33, 0.43, and 0.12 μmol/g, respectively. If the SCFA concentrations were lower than the minimum detection limits of acetate, propionate, and butyrate concentrations, the concentrations were adjusted to 1.32, 0.42, and 0.11 μmol/g, respectively. Due to the different water contents of the individual fecal samples, the fecal SCFA concentrations were reported as μmol/g of fecal dry matter.

### Questionnaires

The online questionnaires were sent to each owner at the beginning and the end of the study. These contained questions about seizure semiology, nutrition, behavior, and cognition based on the formerly validated cBARQ (40), ADHD (41), and CCDR (42) questionnaires.

### Statistics and data analysis

The data were collated and analyzed using Prism® Version 9.3.1 (GraphPad Software, San Diego, CA, USA). The level of significance was set at a *P*-value of <0.05. A one-tailed Wilcoxon matched-paired signed-ranks test was used to compare each alpha diversity parameter, taxonomic levels, genes related to metabolic pathways, qPCR-based DI, SCFA concentrations, and behavioral questionnaires between two-time points. To compare the difference between PB-R and PB-NR groups at D90, a one-tailed Mann–Whitney U test was used for analysis. The same method was also used to compare the results at D0 between both groups in order to rule out any potential biases. Multivariate statistical analysis for beta diversity (analysis of similarity, ANOSIM) was performed using Primer 7 (Plymouth Routines in Multivariate Ecological Research Statistical Software, v7.0.13) (43). Univariate statistics were then performed on alpha diversity and bacterial taxa for all taxonomic levels using JMP Pro 12 (Cary, NC, USA). *P*-values were adjusted for multiple comparisons with Benjamin & Hochberg FDR at a *P*-value of <0.05.

## Results

### Dogs

Twelve dogs with IE Tier II confidence levels (33) were included in the study (Table 1). The signalment of the dogs is summarized in Table 1. The mean age (± standard deviation) of the dogs at the beginning of the study and their age at IE onset were 3.5 ± 2.2 and 2.7 ± 1.7 years, respectively. Of the 12 dogs, 10 dogs were male and two dogs were female. The dog breeds varied, with no breed being overrepresented. The administered dose of PB was 2.3 ± 0.4 mg/kg every 12 h. The PB serum concentrations at D30 and D90 were 19.8 ± 5.3 and 18.1 ± 3.7 mg/L, respectively. After the start of PB treatment, seven dogs were seizure-free, while five dogs had at least one seizure within the 90-day study period. In this study, the dogs experiencing seizure freedom since starting PB were grouped as PB-Responder (PB-R), and the dogs not responding to the initial dose were grouped as PB-Non-Responder (PB-NR).

**Table 1:** Signalment of dogs included in the study, age of IE onset, diets, treats, experience of cluster seizures (CS) or status epilepticus (SE) in the last 90 days before starting phenobarbital (PB), PB concentrations (conc.) 30 days post-treatment (D30), and 90 days post-treatment (D90), responses to PB, seizure frequency (SF) per month during the study, dysbiosis index (DI) at pre-treatment (D0), and D90 of each dog.

**Case number**	**Breed**	**Gender**	**Age (year)**	**Age of IE** ** onset (year)**	**Diets**	**Treats**	**CS/SE** ** before PB**	**PB conc. (mg/L)**		**PB response**	**SF**	**DI**	
								**D30**	**D90**			**D0**	**D90**
1	American Bully	male	2.2	2.2	CDF from chicken	Fish and chicken treats, F, V, M	–	14.8	25.1	PB-NR	3	−2.2	−3.2
2	Labrador Retriever	male	4.7	4.5	CDF+CCF from duck	Sausages from poultry	CS	18.5	22.1	PB-R	0	−1.5	−5.0
3	Peruvian Hairless Dog	male	0.7	0.7	CDF from chicken	Pork, chicken, and rabbit treats, F, V, M	–	19.3	14.7	PB-R	0	0.1	−4.6
4	Poodle	male	6.9	6.2	CDF from beef	F, V	SE	24	24	PB-NR	0.33	−2.8	−4.8
5	Rhodesian Ridgeback	female	3.6	3.6	CDF from duck, lamb, and horse	Pork, beef, and horse treats	CS	33.1	17.6	PB-R	0	5.2	−2.2
6	Australian Shepherd	male	2.8	2.8	CDF from ostrich	V	–	16.8	17	PB-R	0	−0.9	−5.5
7	Labrador Retriever	male	4.9	4	CDF from chicken, pork, lamb, and fish	Beef and chicken treats, eggs, F, V	–	24.5	16.6	PB-R	0	−6.9	−4.8
8	Crossbreed	male	7.8	0.7	CDF from fish	Beef and duck treats, F, M	CS	21	17.3	PB-NR	0.33	−0.3	−5.7
9	Rottweiler	male	3.5	3.5	CDF from duck	–	CS	18	15.4	PB-R	0	−4.3	−3.1
10	Crossbreed	male	1.2	1.2	CDF from lamb	–	–	17.3	17.2	PB-NR	1	−4.9	−5.1
11	French Bulldog	male	3.3	1.3	CDF from chicken	–	–	19.2	13	PB-NR	0.33	−5.4	4.7
12	Dachshund	female	0.7	0.5	CDF+CCF from duck	Beef treats, M	CS	10.9	17.5	PB-R	0	−3.8	−3.5

*CDF, commercial dry food; CCF, commercial canned food; F, fruits; V, vegetables; M, milk products such as cheese and yogurt; PB-R, PB-Responder; PB-NR, PB-Non-Responder*.

### Alpha diversity

**Figure 1:**
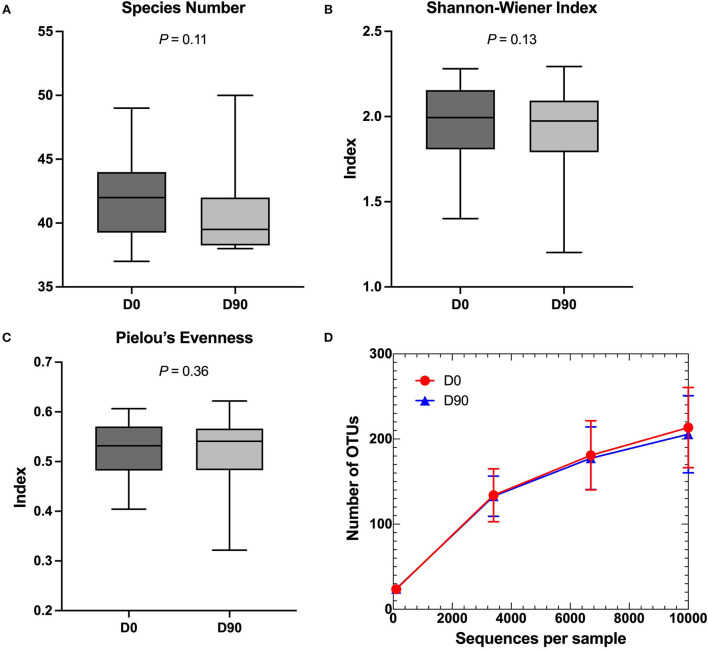
**(A–C)** Comparison of alpha diversity parameters in box and whiskers plots. **(A)** Species number, **(B)** Shannon–Wiener index, and **(C)** Pielou's evenness demonstrated no significant changes between pre-phenobarbital treatment (D0) and 90-day post-treatment (D90). Lines in the boxes represent the median of each group and the boxes represent the interquartile range, whereas the whiskers represent the minimum and maximum data. **(D)** Rarefaction curve based on the number of observed operational taxonomic units (OTUs). The red circles and blue triangles represent the mean of sequences per sample at D0 and D90, respectively. The error bars represent standard deviations.

No differences in alpha diversity parameters, namely, species richness, Shannon–Wiener index, Pielou's evenness, and OTU rarefaction, were found within the samples when comparing the two-time points (D0 and D90) (Figure 1).

### Beta diversity

No significant microbiome clustering between D0 and D90 or between the PB-R and PB-NR group at D90 was observed.

**Figure 2:**
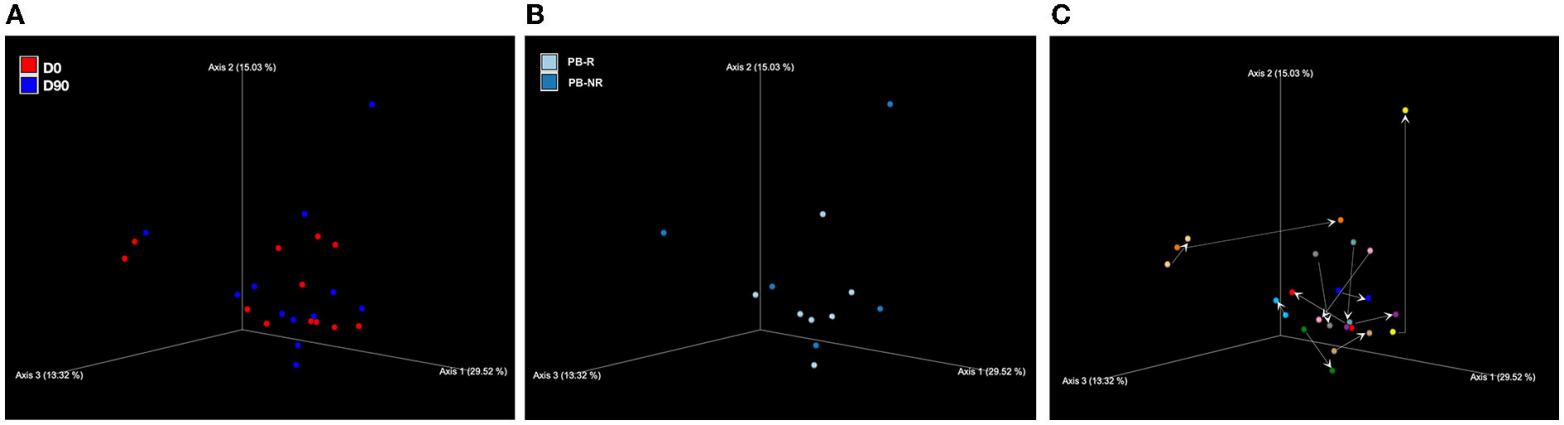
Principal coordinates analysis (PCoA) of weighted UniFrac distances of taxa diagrams demonstrating beta diversity **(A)** between the day prior to phenobarbital (PB) treatment (D0; red) and 90 days post-PB treatment (D90; blue), and **(B)** between PB-R group (light blue) and PB-NR group (dark blue) at D90. There was no significant microbiome clustering. **(C)** Changes in PCoA weighted UniFrac distances of taxa after PB treatment. The arrows show the direction from D0 (arrow tail) to D90 (arrow head) of each dog.

Also, when comparing feces from the PB-R group and PB-NR group sampled at D0, no significant microbiome clustering was detected. There was also no significant difference in beta diversity in the PB-NR group at D90 compared to D0, which was similar to the PB-R group. The beta diversity is shown in Figure 2.

### Taxonomic difference

**Figure 3:**
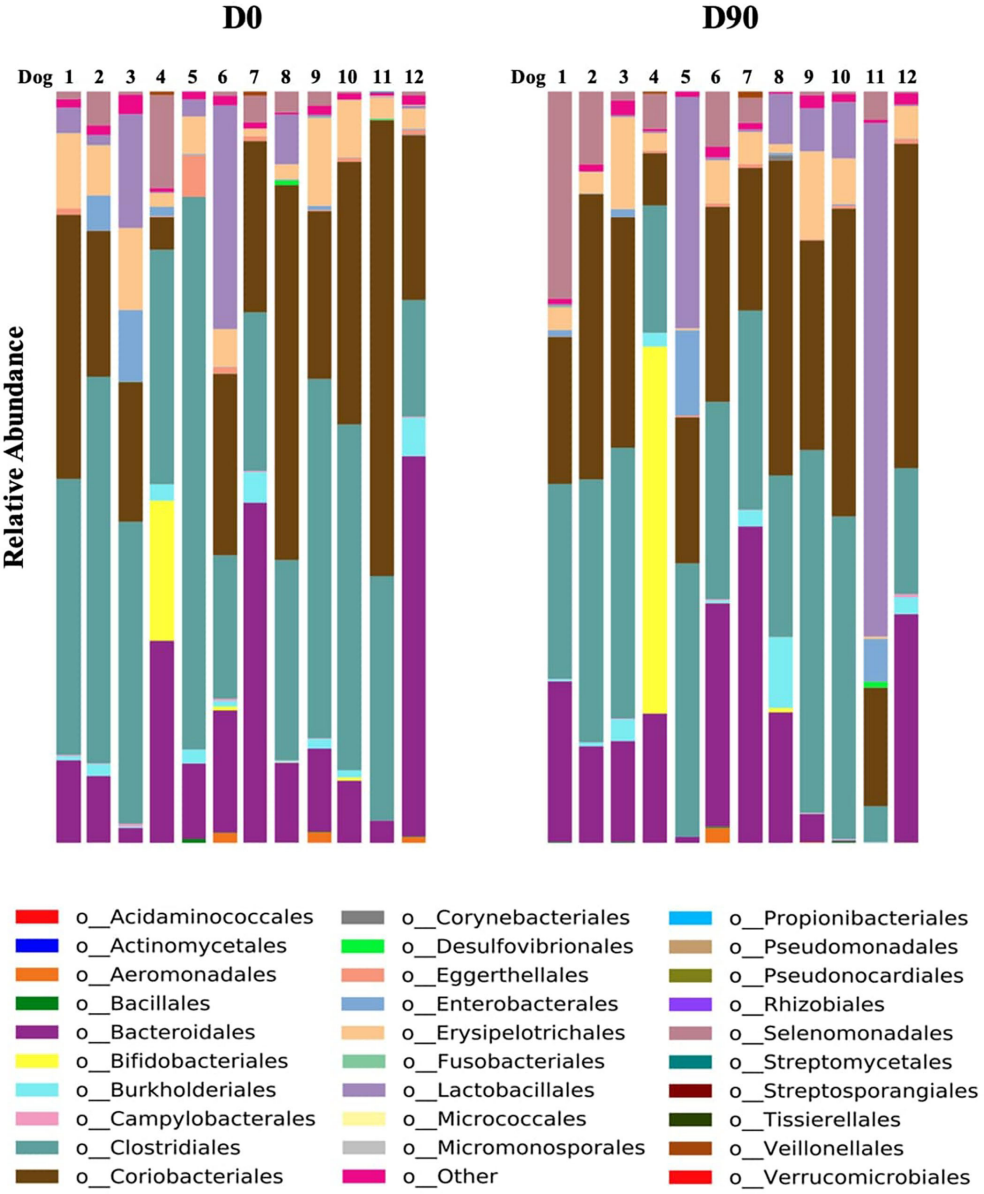
Relative abundance of each order prior to phenobarbital treatment (D0) and 90 days post-treatment (D90) in fecal samples of each dog (*n* = 12).

**Figure 4:**
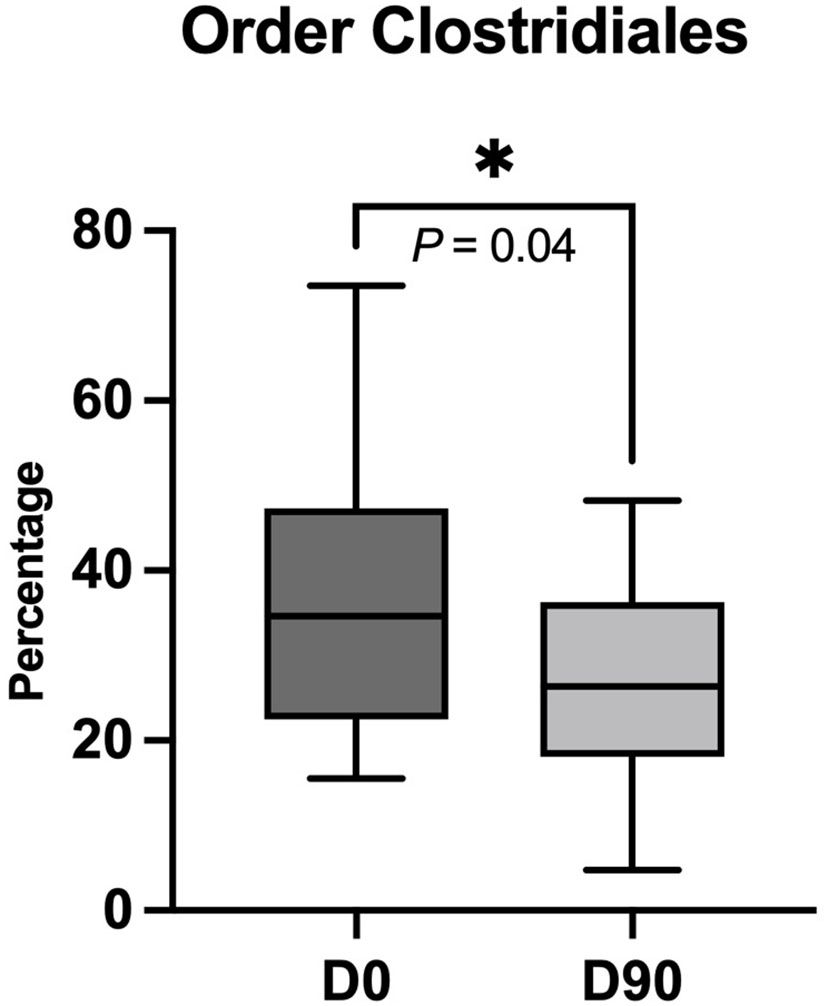
Significant decrease in order Clostridiales in percentage after 90 days of phenobarbital treatment (D90) compared to pre-treatment (D0). Lines in dark and light gray boxes represent the median at D0 and D90, respectively. The box limits indicate the interquartile range, while the whiskers represent the minimum and maximum data. *P < 0.05 (one-tailed Wilcoxon matched-paired signed-ranks test).

The metagenomics by shotgun gene sequencing showed no significant change in fecal bacterial taxa at phylum, family, genus, and species level. There was only a significant decrease in the order Clostridiales at D90 compared to D0 (*P* = 0.04) (Figures 3, 4). The sequence data are deposited in the NCBI Short Read Archive (SRA) database (Accession Number: PRJNA849257).

### Functional genes analysis

The comparisons of the functional genes concentrations measured by metagenomics analysis between D0 and D90 demonstrated a significant increase in the number of microbial ribosome genes (KEGG module M00178, *P* = 0.02), genes associated with the conversion from oxaloacetate to fructose 6-phosphate in the gluconeogenesis pathway (KEGG module M00003*, P* = 0.04), and D-glucose 1-phosphate to trehalose in the trehalose biosynthesis pathway (KEGG module M00565, *P* = 0.04). Additionally, a significant decrease in V-type ATPase genes as part of the oxidative phosphorylation pathway (KEGG module M00159, *P* = 0.01) was evident. The sequence data are deposited in the NCBI SRA database (Accession Number: PRJNA849257).

### Dysbiosis index

In parallel to the sequencing data, the qPCR-based DI comparing D0 and D90 did not show any significant changes (Table 1). At D0 and D90, 10/12 (83%) and 11/12 (92%) dogs had a DI <0, respectively.

### Short-chain fatty acids

**Figure 5:**
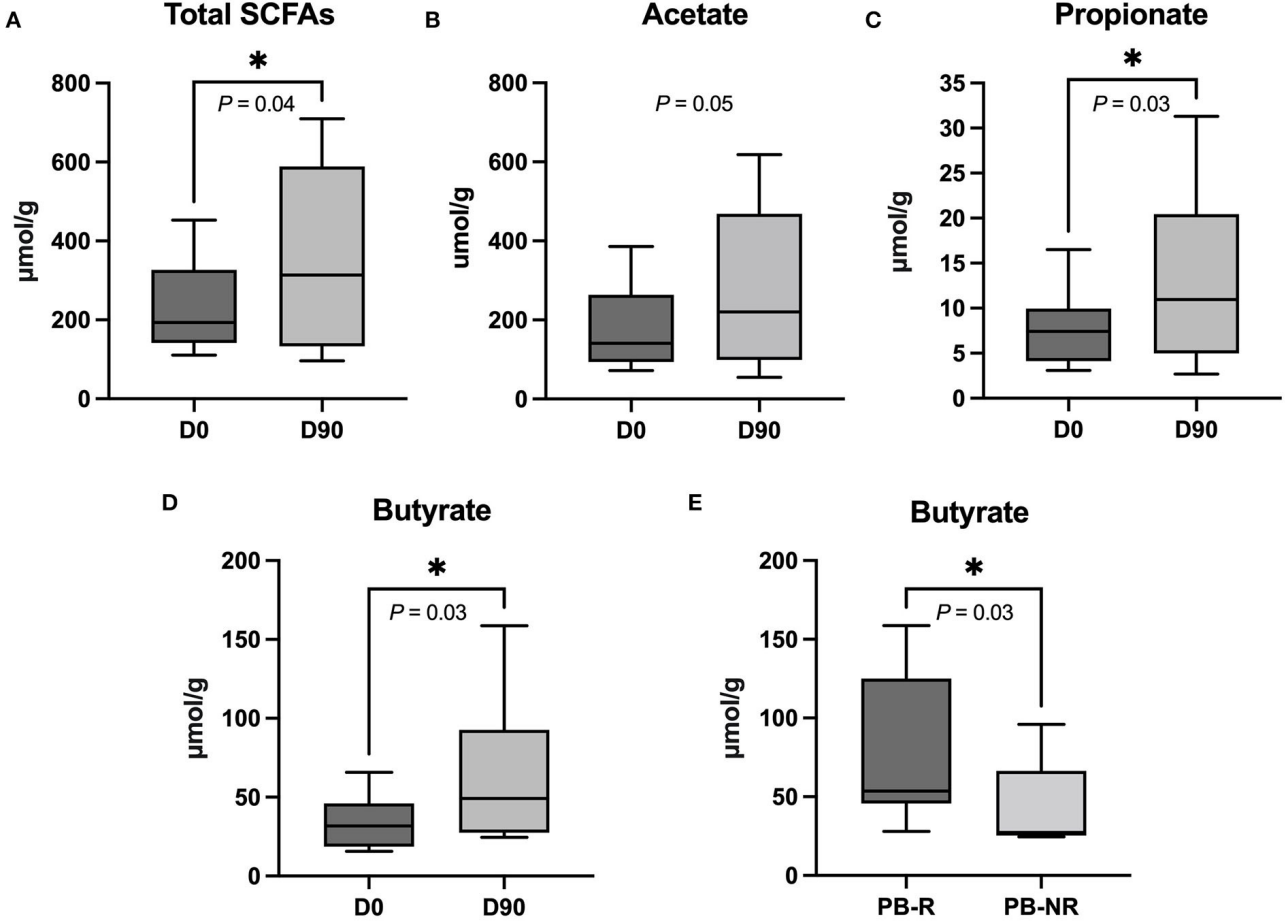
**(A)** The total short-chain fatty acids (SCFAs), **(C)** propionate, and **(D)** butyrate levels in fecal samples increased significantly when comparing 90 days after phenobarbital (PB) treatment (D90) to the day before PB treatment (D0). **(B)** The graph shows an upward trend of acetate at D90 compared to D0. *P < 0.05 (one-tailed Wilcoxon matched-paired signed-ranks test). **(E)** Butyrate level at D90 differed between PB-R and PB-NR. *P < 0.05 (one-tailed Mann–Whitney *U*-test). The lines in the boxes indicate a median value and the box limits represent the interquartile range. The whiskers indicate minimum and maximum data.

The total fecal SCFA concentrations and the concentrations of propionate and butyrate increased significantly from D0 to D90 (*P* = 0.04, 0.03, 0.03, respectively), while acetate concentrations showed a marginal trend but did not reach statistical significance (*P* = 0.05). There were no significant changes in the levels of isobutyrate (*P* = 0.36), isovalerate (*P* = 0.31), and valerate (*P* = 0.16) between both time points. The comparison between the PB-R group and the PB-NR group revealed that the PB-R group had significantly higher concentrations of butyrate (*P* = 0.03). Respective graphs are shown in Figure 5.

### Behavioral analysis

The cBARQ, ADHD, and CCDR values were compared between D0 and D90. The cBARQ results showed a significant decrease in stranger-directed fear (0.20 ± 0.32 [D0] vs. 0.08 ± 0.19 [D90], *P* = 0.03), non-social fear (0.49 ± 0.41 [D0] vs. 0.34 ± 0.26 [D90], *P* = 0.04), and an increase in trainability (0.85 ± 0.44 [D0] vs. 1.01 ± 0.39 [D90], *P* = 0.02). The results showed that stranger-directed fear, non-social fear, and trainability improved after PB treatment. Neither ADHD nor CCDR scores changed between D0 and D90. All CCDR values at D0 (34.91 ± 3.75) and D90 (35.75 ± 6.73) were under the score of 50, below the canine cognitive dysfunction (CCD) score (32). Comparing each parameter between the PB-R group and PB-NR group revealed no significant differences.

## Discussion

PB as the primary drug of choice has a long history in canine IE treatment. However, its role in behavior and GIM has not been fully elucidated. PB improved stranger-directed fear, non-social fear, and trainability. Apart from the abundance of the bacterial order Clostridiales being reduced after PB treatment, no other GIM changes could be demonstrated. The fecal DI indicated that 92% of the dogs were within the reference interval established for healthy dogs. The functional metagenomics analysis mainly demonstrated changes in protein synthesis and glucose metabolism pathways, whereas there was an increase in the total SCFAs, butyrate, and propionate.

Similar to the current study, another study in nine dogs showed no changes in the alpha and beta diversity of GIM from fecal samples of drug-naïve IE dogs compared to 30 days after PB- or imepitoin treatment (15). However, the dosage of PB might play a role here. In a preclinical study, the effects of different PB concentrations on several bacteria, namely, Bifidobacterium, Bacteroides, Enterococcus, Eubacterium, Clostridium, and Staphylococcus were tested *in vitro*. The study found that generation time and lag time during the growth of some bacteria were affected depending on PB concentrations, while the lag time was influenced only by a high concentration of PB. Additionally, the metabolite alpha-ethyl-benzeneacetamide was found as a by-product of Bifidobacterium in both low and high PB doses (44). One of the metabolite synonyms is 2-phenylbutyramide, which was shown in several studies in mice as having a promising antiseizure effect by inhibiting neuronal acetylcholine receptors (45, 46). In the present study, a starting dosage of PB was used (2, 3). Higher PB concentrations might have yielded a different effect and produced a similar effect *in vivo* as seen *in vitro* in terms of how bacteria are affected.

In the study by Olson and colleagues in mice, *Akkermansia* and *Parabacteroides* bacteria were considered important for seizure control by increasing the seizure threshold (13). In contrast, no genus *Akkermansia* was detected in the current study by metagenomics analysis, while *Parabacteroides* accounted for only 0.1% of sequencing reads in this study. It is worth noting that *Akkermansia* populates mainly the enteric crypts in dogs, so they might not shed easily into feces (47). To investigate this hypothesis, an intestinal biopsy could provide more information. In another canine study, *Bacteroidaceae* species within genus 5-7N15 were more abundant in dogs fed with a medium-chain triglyceride diet (16). These bacteria have previously been suggested to prevent aggressive behavior and occupy a similar niche in humans as *Akkermansia*.

In our study, there was a significant decrease in Clostridiales after PB treatment, which inversely correlates with the SCFA concentration results. Clostridiales are a complex bacterial order, namely, SCFA producers, pathogens, and many others. It is most likely that there were some changes in another Clostridiales member in lower taxa, other than SCFA-producing bacteria, which caused an overall decrease in this order abundance. In the aforementioned study by García-Belenguer and colleagues, the reduction in SCFA-producing bacteria at the family level was reported after 30 days of PB- or imepitoin treatment. Nevertheless, the SCFA concentration was not measured in that study (15).

The concentration of total SCFAs, propionate, butyrate, and also to some extent, acetate, did increase with PB treatment in our study. This is supported by the results of a study by Xie and colleagues (48). The researchers investigated the effects of oral GABA administration on different concentrations of SCFAs in mice and found that GABA could increase the production of total SCFAs, acetate, propionate, and butyrate as well as increase acidity in the intestine. Both PB and GABA work on the same receptor, which is the GABA_A_ receptor (48). However, the reason why PB increases the SCFA production of GIM is unknown. SCFAs are essential for gut and brain health. Due to their being small molecules of fatty acids, they can be rapidly absorbed and transported *via* circulation to the liver and other organs, namely, the brain, easily crossing the blood–brain barrier (BBB) (49). In another study, serum saturated fatty acid concentrations in PB-treated dogs were evaluated and were significantly higher than in controls (50). The beneficial functions of SCFAs are well studied in the intestine but only to a lesser degree in CNS. SCFAs have several positive effects that could potentially support brain functions and palliate epilepsy through MGBA. One of the well-known functions of SCFAs, particularly butyrate, is their anti-inflammatory effect (23, 51, 52). Inflammation plays an important role, especially in drug-resistant epilepsy (53, 54). Nonetheless, whether butyrate can alter brain inflammation in epilepsy requires further studies. However, a study in mice showed that intestinal inflammation could increase convulsant activity and reduce ASD efficiency (55). Furthermore, epilepsy is associated with a change in glucose metabolism (56). Butyrate can provide an alternative energy resource for the brain (57), resulting therefore in a potential improvement in epilepsy. This could be similar to a ketogenic diet in humans and dietary medium-chain triglyceride in canine IE management, where ketones provide an alternative source of energy (56). Butyrate also has neuroprotective effects, which can be explained in several dimensions, such as the ability of neurogenesis stimulation (58), prevention of neuronal apoptosis (59), suppressed demyelination, and promoted remyelination (60), BBB permeability restoration (61), and antioxidation (25). A couple of preclinical studies in rodent models also found that butyrate may have direct antiseizure effects through the histone deacetylase pathway (62, 63). In our study, the butyrate level after PB treatment was significantly higher in the PB-R group compared to the PB-NR group. Nevertheless, it should be taken into account that this study had a small sample size in both groups, which might influence the results. A future study with a larger sample size would be needed to confirm these results. Further studies might also consider adding butyrate to a dog's diet, either directly or indirectly by modifying the microbiome (probiotic or high fiber diet) (57, 64), to see whether this can improve epilepsy management.

Regarding the functional aspect of GIM, we also found that PB treatment could increase the number of genes associated with protein synthesis and carbohydrate metabolism. However, it appears that the GIM under PB treatment was under stress conditions. There are several conditions that could be stressful for GIM, such as osmotic stress, oxidative stress, and acid stress from their SCFA fermentation (65–67). To survive under these conditions, GIM have stress defense mechanisms, well-known ones such as trehalose biosynthesis and transcriptional regulation (65). Interestingly, we found that there was an increased abundance of genes associated with the trehalose biosynthesis pathway during metabolism from D-glucose 1-phosphate to trehalose (KEGG module M00565). Bacterial trehalose biosynthesis typically occurs under abiotic stress in order to increase trehalose concentration (66, 68). On the one hand, the elevation of trehalose concentration nourishes the GIM themselves (66). On the other hand, the accumulation of trehalose results in bacterial rehydration without cellular function deterioration by reducing water activity, cell volume restoration, and stabilizing protein as well as cellular turgor pressure (65). Other than surviving stress conditions by trehalose biosynthesis, an increased number of ribosomes are also essential. In the present study, the abundance of genes related to microbial ribosomal production (KEGG module M00178) was significantly increased, which potentially resulted in an increased number of microbial ribosomes. As mentioned above, transcriptional regulation is also necessary for GIM to alleviate stress conditions. The defense strategies of bacteria are generally “programmed” in specific genes. The responses depend on the type of stress the GIM are facing and the transcription is regulated to react appropriately to each condition. However, the success of transcriptional regulation is determined by the subsequent ribosomal translation, the number and speed of the ribosomes playing a crucial role in this process (69, 70). Since ribosomes are an important factor in bacterial protein synthesis, increasing the ribosome number is not only important for stress responses but also for viability and their protein-related productivity (71). Other elevated gene numbers detected in this study were genes associated with pathways in gluconeogenesis during the metabolism of oxaloacetate to fructose 6-phosphate (KEGG module M00003). GIM normally require glucose as the main carbon source for their metabolism, which they normally uptake from the surrounding environment. When glucose is insufficient, the GIM use gluconeogenesis as an alternative method to synthesize glucose from non-sugar substrates, namely, amino acids and intermediates from the tricarboxylic acid cycle (72). In contrast, V-type ATPase genes as part of oxidative phosphorylation decreased in this study. The V-type ATPase gene is located in the bacterial cytoplasmic membrane and functions as an ATP hydrolysis-driven ion pump, which relates to several GIM processes, namely, intracellular pH regulation and neurotransmitters release (73). However, it is still unclear how the reduction in the V-type ATPase gene might affect this situation. Furthermore, the alterations of the four genes associated with the KEGG modules (M00003, M00159, M00178, and M00565) showed no relation to SCFA production. In our study, we compared the results before and after PB treatment. Each dog received the same diet throughout the study period. Since the diet is one of the important factors affecting GIM (18) as is the dog breed (74), it is worth noting that the lack of dietary and breed standardization in this study might have affected the results. In addition, an age, breed, and diet matched healthy control group could have also added value to the interpretation of the study results.

Fear- and anxiety-related behaviors as well as cognitive dysfunction are well-known epilepsy comorbidities in dogs (26, 27, 32). To date, it has not yet been completely elucidated whether these epilepsy comorbidities are a risk factor for the development of epilepsy, part of the epilepsy phenotype, a side effect of some ASDs, or a combination of all. Our study is the first prospective study focusing on the effect of PB on behavior in dogs with IE. For behavioral assessment in this study, the positive effects of PB were observed by improving stranger-directed and non-social fear as well as trainability. There was no significant difference between the PB-R group and the PB-NR group. These results might appear surprising at first sight, as the opposite is more likely to have been predicted. However, prior to our study, there had been no behavioral longitudinal studies in either animals or humans with epilepsy treated with PB as monotherapy (26). In humans, PB has been used not only to treat seizures but also to treat anxiety (75). Nonetheless, in a small cross-sectional study, it was shown that people with epilepsy treated with primidone, which gets metabolized to PB, were more likely to show anxiety (76). Similar single reports on anxiety-related behavioral changes seen in dogs with IE exist for primidone, but not for PB (77). The influence of PB on cognition remains unclear, with original studies in humans even reporting a potential improvement (75). In dogs with IE, cross-sectional studies did not reveal an improvement or a deterioration in fear/anxiety or cognitive impairment with PB monotherapy (27, 78, 79). Potential improvement in terms of behavior, but also deterioration in individual cases after treatment with individual drugs have been shown for levetiracetam (80) and imepitoin (77). For other drugs, however, this link is not as clear. Nonetheless, aggression as a side effect, which is often idiosyncratic in nature, as seen in individual cases treated with PB, potassium bromide, levetiracetam, and/or zonisamide, has been reported in the literature (77, 81). In addition, polypharmacy and drug resistance have also been associated with cognitive impairments and fear/anxiety, especially in dogs with IE treated *via* monotherapy (27, 32, 79). Interestingly, in one of these studies, pharmacoresistance was an influencing factor, but an association with seizure frequency could not be found (79). This further highlights that the severity of epilepsy is not only reflected by its seizure frequency but also by the severity of its comorbidities. With regard to the influence of ASDs on cognition, Packer and colleagues reported that the trainability of adult dogs with IE was significantly worse compared to controls when dogs received potassium bromide, zonisamide, or polypharmacy (32). The current evidence in canines rather suggests that epilepsy is one of the main factors triggering the development of fear/anxiety and cognitive deficits (27, 29, 32, 78, 79). It is worth mentioning that the diagnostic methods for anxiety and cognitive dysfunction are complex. In humans, the gold standard of anxiety disorder diagnosis is a clinical interview by a specialist (82), while mild cognitive impairment is normally assessed using the Montreal Cognitive Assessment and Mini-Mental State Examination (83). To date, there is a paucity of data showing that self-reported or symptom-based questionnaires could replace the gold standard (82). In dogs, diagnoses of fear- and anxiety-related behaviors and cognitive dysfunction are as complicated as in humans and the gold standards have not been established. Therefore, history taking and completion of validated questionnaires by dog owners as well as the performance of dog behavioral tests or observations and behavioral development assessments are used in combination as diagnostic procedures (84, 85). In the present study, only validated online questionnaires were used to evaluate the behavioral comorbidities. This should be kept in mind, when considering the results, as it is a limitation of the current study.

It is possible that the beneficial effects on stranger-directed and non-social fear-related behavior and trainability after PB treatment may be due to elevated SCFAs, particularly the butyrate level (57). Such effects were also shown in human cases and mice models receiving a high fiber diet, modulating the GIM to produce these SCFAs (86, 87). An improvement in anxiety-like behavior and cognitive impairment was shown in a number of studies on sodium butyrate, a salt form of butyric acid in rodent models (88–90). Sodium butyrate is also a histone deacetylase inhibitor and was reported to be a potential anti-depressant, inducing short-lasting histone hyperacetylation in the hippocampus and frontal cortex as well as temporarily increasing brain-derived neurotrophic factor (BDNF) expression in mice (91). Not only in preclinical studies was the inverse correlation between butyrate concentration and the degree of anxiety observed but also in humans with anorexia nervosa (92), which agrees with our findings. In our study, improvement in trainability without alleviating cognitive ability was observed, which could be explained by its multifactorial correlation. In dogs, trainability is a combination of motivation and willingness to follow the commands of their owners, difficulty in being distracted, and positive reaction to correction as well as cognitive skills (40, 93). Therefore, the improvement in fear- and anxiety behaviors could also play a positive role in trainability.

## Conclusion

PB treatment in canine IE could affect GIM taxonomically and functionally including an increase in SCFAs level as well as altering glucose and protein metabolism of GIM by increasing the abundance of their functional genes. In addition, the level of butyrate among PB-R was significantly higher than that of PB-NR, showing that butyrate could be essential and beneficial for seizure control. This could be explained by providing an alternative efficient energy source and by the anti-inflammatory and neuroprotective effects of butyrate. Moreover, PB treatment may have the potential to alleviate fear- and anxiety-related behaviors as well as trainability in dogs with IE. Future large-scale studies including consulting a behavior specialist are needed to confirm these results.

These findings could be an important first milestone showing that the difference in microbial functional reactions could be the key difference between the effective and ineffective treatment of dogs with epilepsy. More studies on GIM functions and their role in epilepsy management, as well as behavioral comorbidities, are needed to better our understanding and to consider the development of alternative effective treatment strategies in canine IE.

## Data availability statement

The datasets presented in this study can be found in online repositories. The names of the repository/repositories and accession number(s) can be found below: SRA data: PRJNA849257/Temporary Submission ID: SUB11612854.

## Ethics statement

Ethical review and approval was not required for the animal study because all dogs in this study received the standard of care, therapeutic and diagnostic work-up. The fecal samples were collected after voiding. Written informed consent was obtained from the owners for the participation of their animals in this study.

## Author contributions

AW planned and performed this study as well as interpreted the results and drafted the manuscript as a part of her Ph.D. thesis. SM and HV planned, co-supervised, and reviewed the study. JS, MK, and RP performed the laboratory part and supervised and reviewed the study. SL, LB, and AB-N were responsible for some dogs from their own institutes and reviewed the study. GM-W supervised and reviewed the study. All authors contributed to the article and approved the submitted version.

## Funding

This open access publication was funded by the Deutsche Forschungsgemeinschaft (DFG, German Research Foundation) – 491094227 – Open Access Publication Funding and the University of Veterinary Medicine Hannover, Foundation. AW is a holder of a scholarship from the Faculty of Veterinary Medicine, Kasetsart University, Thailand.

## Conflict of interest

Author HV served as a paid consultant in the field of epilepsy for Boehringer Ingelheim, CEVA Animal Health, Nestle Purina, and as a contract researcher for Nestle Purina, Desitin Pharma, and Boehringer Ingelheim. Author JS is an employee of the Gastrointestinal Laboratory at Texas A&M University that provides microbiome testing on a fee-for-service basis and is on the scientific advisory board for Nestle Purina and received speaker honoraria from Royal Canin, Nutramax Laboratories, ExeGi Pharma, LLC, and Hill's Pet Nutrition, Inc. The remaining authors declare that the research was conducted in the absence of any commercial or financial relationships that could be construed as a potential conflict of interest.

## Publisher's note

All claims expressed in this article are solely those of the authors and do not necessarily represent those of their affiliated organizations, or those of the publisher, the editors and the reviewers. Any product that may be evaluated in this article, or claim that may be made by its manufacturer, is not guaranteed or endorsed by the publisher.
